# Axial Tensile Adhesively Bonded Performance of Carbon Fiber Composite Tubes Under Room-Temperature and Low-Temperature Circulation

**DOI:** 10.3390/ma18051124

**Published:** 2025-03-02

**Authors:** Haibo Luo, Qian Wang, Yanchu Yang, Tao Li, Jun Wu, Wentao Gong, Hui Feng, Xiaohui He

**Affiliations:** 1 Key Laboratory of Earth Observation of Hainan Province, Hainan Aerospace Information Research Institute, Wenchang 571300, China; 2 Aerospace Information Research Institute, Chinese Academy of Sciences, Beijing 100094, China

**Keywords:** composite circular tube, titanium alloy tube, thermal cycling (room-to-low-to-room-temperature), adhesive bonding performance, experimental study

## Abstract

This study investigated the axial tensile performance of adhesively bonded T700/C204 carbon fiber composite and TC4 titanium alloy tubular single-lap joints under three distinct temperature conditions: room temperature, low temperature (−65 °C), and room–low–room-temperature cycling. Two configurations of adhesively bonded joints—composite–composite and composite–titanium—were tested. Specimens were designed to evaluate the influence of spew-fillet and perfect lap configurations on uniaxial tensile bonding strength across varying temperature environments. Analysis of the final failure morphology, stress concentration locations, ultimate failure loads, and load-displacement curves revealed that stress concentration and peeling stress were most pronounced at the ends of the bonded region, which served as the initiation points for failure. The adhesively bonded joints exhibited two distinct failure modes, strongly correlated with material properties and environmental temperature. The titanium alloy tubular joints predominantly experienced adhesive layer failure, while the carbon fiber three-way tubular joints were primarily characterized by fiber-tear failure. Environmental temperature significantly influenced the strength of the adhesively bonded joints. Specifically, the tensile failure limit of the bonded specimens subjected to low-temperature cycling (25~−65~25 °C) was approximately 61% higher than that observed under the room or low-temperature conditions. Furthermore, the experimental results demonstrated that a maximum failure load of 27.522 kN and a shear strength of 10.956 MPa were achieved. Notably, the presence of adhesive spew-fillet had a negligible impact on the bonding strength of the joints.

## 1. Introduction

Connections between composite structures and between composites and metal structural parts have been widely utilized in the fields of aerospace, nuclear industry, and marine engineering. Compared with the traditional mechanical connection, the adhesively bonded connection is characterized by lighter weight, higher connecting efficiency, better damage safety, better damping, better insulation performance, and smoother aerodynamic shape. As such, adhesively bonded connections are being increasingly utilized for connections between composite structures and between composites and metal structural parts. Single-lap joints are widely used in engineering because they offer several advantages, such as simplicity of technique, good economic value, high connection efficiency, and good adhesively bonded connection. Thus, the single-lap joint of composite materials [1,2,3,4] has drawn considerable attention from scholars. Some studies [5,6,7,8] have shown that temperature variation has a great influence on the mechanical properties of composite materials; however, few studies have reported on the specific application of composite tube single-lap joints in the lightweight structure of near-space aerostat at various temperature ranges (−65~25 °C).

Compared with the adhesively bonded joint between metals, composite materials exhibit anisotropy, low interlaminar strength, and are complex adhesively bonded, among other characteristics. The adhesively bonded failure mode is closely related to various factors, including lap length, adhesive layer thickness, and ply sequence. Scholars [9,10,11,12,13,14,15,16] have explored adhesively bonded theory since the 1930s. Moura et al. [17] and Anyfantis et al. [18] evaluated the mechanical behavior and failure modes of single-lap joints by using the finite element method. Luo et al. [19] conducted a progressive failure study on the T700/TDE86 composite single-lap joint based on the cohesive zone model, and their results showed that the maximum normal and shear stresses appeared at the two edges along the lap width. Li et al. [20] performed comprehensive experiments on three types of adhesively bonded joints. Their results indicated that under the same parameters, the double-lap joint achieved the maximum ultimate failure load and the lap joint attained the maximum shear strength. In recent years, scholars have investigated the performance of double-lap joints [21,22,23,24,25,26,27] and achieved significant results. Li et al. [28] studied the interlaminar shear strength of adhesively bonded carbon/carbon composites, it was found that the adhesive is quite effective in enhancing the interlaminar strength of the C/Cs, and the shear stress $\tau_{13}$ is the principal factor for the damage. However, few experimental studies have reported reliable and effective adhesively bonded tubular joints concerning different temperatures, failure modes, failure morphologies, stress concentration modes, and ultimate load under different parameters. Although many studies have considered the adhesively bonded joint between composite laminates [29,30,31,32,33], limited works are available on the performance of adhesively bonded lap joints between the composite tube and between the titanium and composite tube.

Recently, M. Shishesaz et al. [34] conducted a study focusing on the impact of adhesive defects, adherend types, and materials on stress distribution within laminated composite tubular joints subjected to axial tensile loads. In a related investigation, Isaiah Kaiser et al. [35] explored the mechanical behavior and failure mechanisms of thin-walled carbon fiber-reinforced polymer (CFRP) and titanium adhesive tubular lap-joints (TLJs) across a range of temperatures, including cold, room, and elevated conditions. Nicolas et al. [36] examined the effect of geometric parameters on the strength of hybrid CFRP-aluminium tubular adhesive joints, revealing that joint strength markedly increases with the bonding area, albeit with a critical overlap length beyond which no further strength enhancement is observed. Higgoda et al. [37] analyzed the structural performance of innovative non-metallic pultruded circular tubular GFRP T-joints under axial compression, advocating for their application in future offshore tubular structure constructions. Mohsen Barzegar et al. [38] utilized the cohesive zone method to assess failure and stress distribution in the adhesive region of a composite T-joint under bending load conditions. Additionally, Luo et al. [39,40] proposed an innovative lightweight and high-strength connection method for three-dimensional braided composite circular tube structures, achieving an impressive load-bearing capacity exceeding 120 kN. This method holds significant engineering value and provides core technical support for the lightweight design and heavy-load capacity of near-space aerostat structures.

This study aims to explore the mechanical characteristics of adhesively bonded T700/C204 composite circular tube single-lap joints, as well as single-lap joints between composite circular tubes and TC4 titanium alloy, under room temperature, low temperature (−65 °C), and room–low–room-temperature cycling conditions. Initially, an experimental investigation was conducted to evaluate the tensile strength of composite–composite and composite–titanium tubular single-lap joints at both room and low temperatures. The failure morphology, ultimate load, and failure mechanisms were thoroughly analyzed. Subsequently, the mechanical properties of the composite circular tubes and titanium alloy tubes were examined, with a focus on assessing the effects of spew-fillet geometry, ideal perfect adhesive bonding, and low-temperature cycling on the tensile failure mechanisms. Finally, the ultimate load, tensile strength, and shear strength were quantified to provide valuable insights for the application of carbon fiber composite adhesively bonded joints in the pod structures of a near-space aerostat.

## 2. Experimental Study

### 2.1. Specimen Details

**Figure 1:**
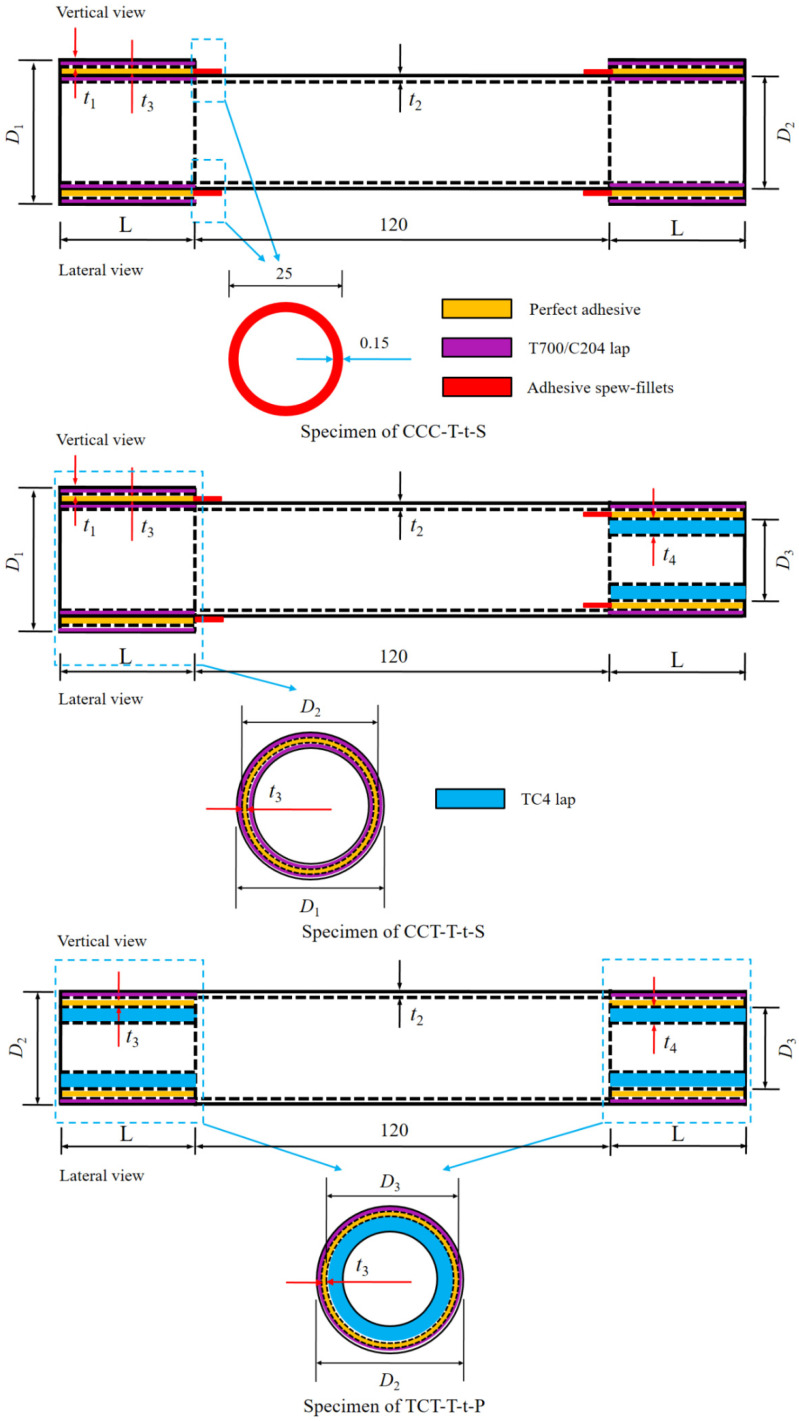
Details of the test specimens (unit: mm).

The physical dimensions of the three types of test specimens are illustrated in Figure 1. All adhesively bonded specimens had an effective length of 200 mm. The carbon fiber three-way tube featured a thickness of 2.5 mm (t1). The composite circular tube had an outer diameter of 25 mm (D2) and a wall thickness of 2.5 mm (t2). The titanium alloy tubular joint, designed as a hollow shell structure, exhibited an outer diameter of 20 mm (D3) and a wall thickness of 4 mm (t4). The outer surface of the TC4 titanium alloy joint underwent sandblasting treatment. All test specimens were configured with a lap length of 40 mm (L). The composite tube was fabricated using carbon fiber/epoxy unidirectional tape (T700/C204, Toray Industries, Tokyo, Japan), while the titanium alloy tubular component was made of TC4 (Bozhong Metal Group, Shanghai, China). The bonding process utilized an epoxy resin film (J-250, Heilongjiang Institute of Petrochemistry, Harbin, China) with a thickness of ±0.15 mm (t3). The relevant material properties of the composites are detailed in Table 1 [41].

The specimens were fabricated through the following procedures: first, the composite tubes were heated to 145 °C for 2 h and then to 185 °C for 3 h at a heating rate of 2 °C/min. During this process, a pressure of 0.5 MPa was maintained in an autoclave for 3 h. Subsequently, the adherend and epoxy resin film, along with both end-laps, were cured at 100 °C for 3 h under a pressure of 0.3 MPa. Following the curing process, the single-lap joints were cut into specimens with the appropriate dimensions for axial tensile testing.

**Figure 2:**
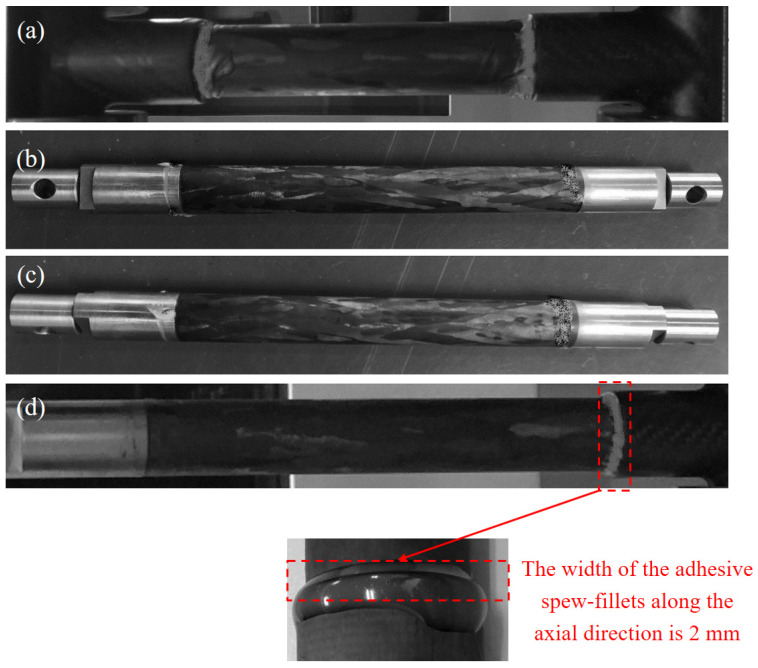
Single-lap joint test specimens: (**a**) adhesive spew-fillets, 25 °C; (**b**) perfect adhesive, 25~−65~25 °C. (**c**) Adhesive spew-fillets, 25~−65~25 °C; (**d**) adhesive spew-fillets, −65 °C.

Two types of single-lap joints were selected for failure testing. The specifications of the test specimens are detailed in Table 2. To ensure the validity of the uniaxial tensile tests, three repeated specimens were prepared for each joint type to achieve reliable experimental results. The ply sequence for both the composite circular tube and the carbon fiber three-way tube was [0/±45/0]_2S_, with each single layer having a thickness of 0.156 mm. Failure tests were conducted on each type of single-lap joint (Figure 2) under different testing conditions: room-temperature, low-temperature (−65 °C), and room–low–room-temperature cycling. The joints were prepared with either a spew-fillet or a perfect adhesive bonding configuration.

For ease of identification, the test specimens were labeled using the format CCC/CCT/TCT-T-t-P/S. Specifically, the labels were as follows:

- CCC-T-t-S specimens consisted of two carbon fiber three-way tubes adhesively bonded at both ends.
- CCT-T-t-S specimens featured a carbon fiber three-way tube adhesively bonded at one end and a titanium alloy tube at the other end.
- TCT-T-t-P/S specimens comprised two titanium alloy tubes adhesively bonded at both ends.

In the labeling system:

- T represents the average thickness of the adhesively bonded circular tube.
- t denotes the thickness of the adhesive layer.
- P indicates a perfect lap joint.
- S refers to the adhesive configuration with a spew-fillet at the end of the lap region.

An example of the CCT-T-t-S joint with a spew-fillet is illustrated in Figure 1. The spew-fillet width along the lap length was 2 mm.

### 2.2. Test Method

A quasi-static axial displacement tensile failure test was performed on all specimens using a universal electronic testing machine (INSTRON 5969, Norwood, MA, USA). Given that the flight altitude of an aerostat is approximately 20 km, where the operating temperature of the composite adhesively bonded joints is around −50 °C, and considering the temperature variation range of −65 °C to 25 °C as the near-space aerostat ascends from ground level to stratospheric altitude, the composite circular tube adhesively bonded joints in this study were tested under these varying temperature conditions.

**Figure 3:**
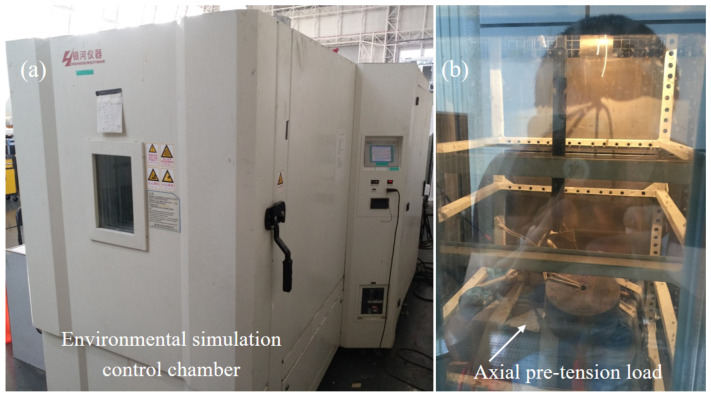
Pre-tension at one end of the test specimen. (**a**) Environmental simulation chamber, (**b**) The sample is inside the environmental simulation chamber.

At room temperature, the test specimen, equipped with an installed clamp, was directly mounted onto the testing machine for evaluation. For low-temperature testing (−65 °C), the specimen was first placed in a low-temperature control box. Liquid nitrogen was then introduced into the box using a cryogenic dewar, gradually reducing the environmental temperature from room temperature to −65 °C. After a stabilization period of 15 min, the displacement load tensile test was conducted. Under environmental simulation cycling conditions (25 °C to −65 °C to 25 °C), a lead weight was attached to one end of the specimen (Figure 3), and a pre-tension load of 850 N was maintained for 12 h in the environmental simulation control box. This included 1.5 h for cooling from 25 °C to −65 °C, 9 h at −65 °C, and 1.5 h for warming back to 25 °C. Following this, the specimen was removed and allowed to stabilize at room temperature for 24 h before being mounted onto the mechanical testing machine for the tensile failure test. A consistent loading rate of 1 mm/min was applied to all specimens, adhering to the ASTM D5868-95 standard [42]. The progressive failure of the specimen was recorded using a digital camera, while the corresponding crosshead tensile load–displacement curve data were captured using a dynamic signal testing system (DH3816N, Donghua Group, Taizhou, China).

## 3. Test Results and Discussions

### 3.1. Influence of Failure Modes at Room Temperature

**Figure 4:**
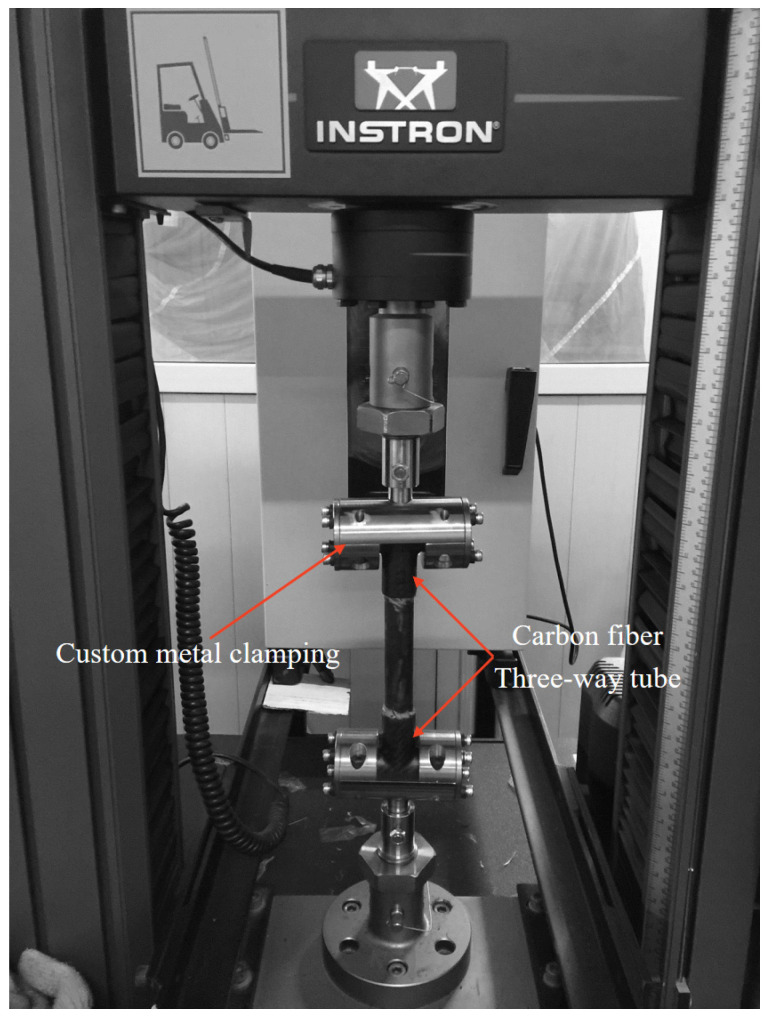
Schematic diagram of the custom metal clamping fixture.

The effect of perfect adhesive bonding and spew-fillet configurations on the performance of adhesively bonded axial single-lap joints was assessed at room temperature. To facilitate the loading process during testing, the carbon fiber joint was designed in a three-way tube configuration. A custom metal clamping (Figure 4) fixture was developed and securely mounted onto the universal electronic testing machine.

**Figure 5:**
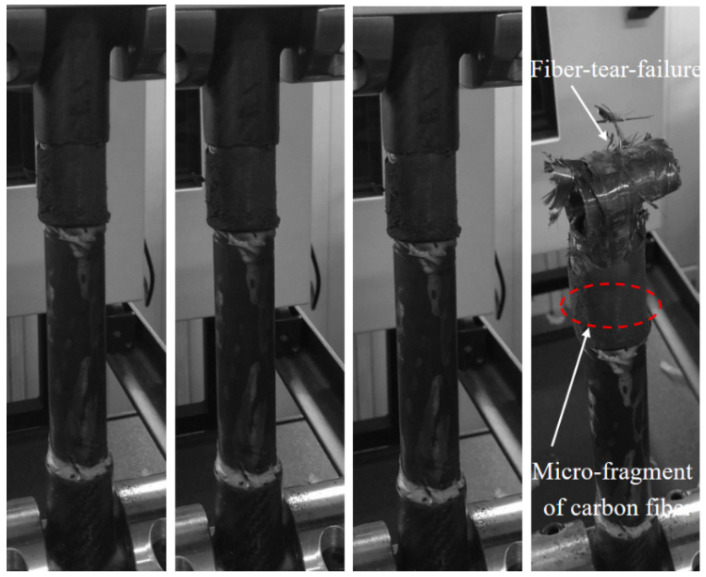
Progressive failure process and final failure morphology of the CCC-T-t-S joint.

Figure 5 illustrates the ultimate failure morphology of the CCC-T-t- S joint, predominantly characterized by fiber-tear failure in the carbon fiber three-way tube. Observations revealed that the carbon fiber three-way tube at the specimen’s upper end initially underwent fiber-tear failure. This was followed by a progressive failure process, culminating in the complete loss of the joint’s load-bearing capacity. At the termination of the overlap, localized cracks emerged at the end of the three-way tube due to stress concentration. The ultimate failure mode manifested as end fiber-tear, accompanied by the presence of carbon fiber micro-fragments. Within the three-way tube failure mode, the final tensile failure load was significantly correlated with the strength of the three-way tube.

**Figure 6:**
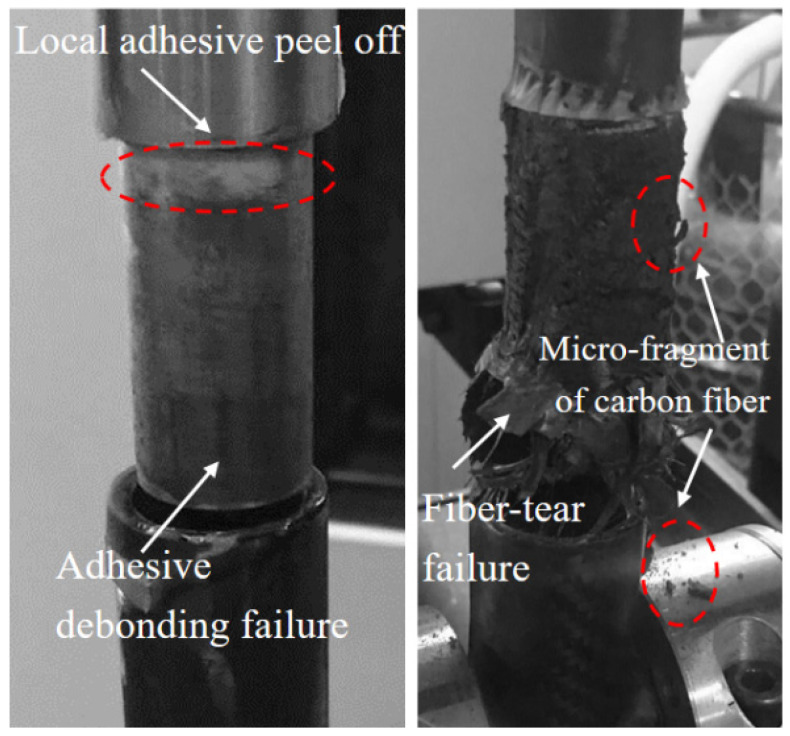
Final failure morphology of the CCT-T-t-S joint.

Figure 6 depicts the final failure morphology of the CCT-T-t-S joint, primarily characterized by fiber-tear failure in the carbon fiber three-way tube and adhesive debonding failure in the titanium alloy tube. It was observed that the carbon fiber three-way tube at the specimen’s bottom end initially experienced fiber-tear failure, followed by adhesive debonding failure at the titanium alloy tube on the upper end, ultimately leading to the complete loss of the joint’s load-bearing capacity. Local adhesive peeling was noted on the surface of the titanium alloy tube wall, with the most severe peeling occurring on both sides of the metal tube. A comparison of the failure modes in the two lap regions revealed that the primary factor influencing the titanium alloy tube failure was the shear failure strength of the adhesive, while the fiber tensile failure strength was the dominant factor affecting the carbon fiber three-way tube failure.

**Figure 7:**
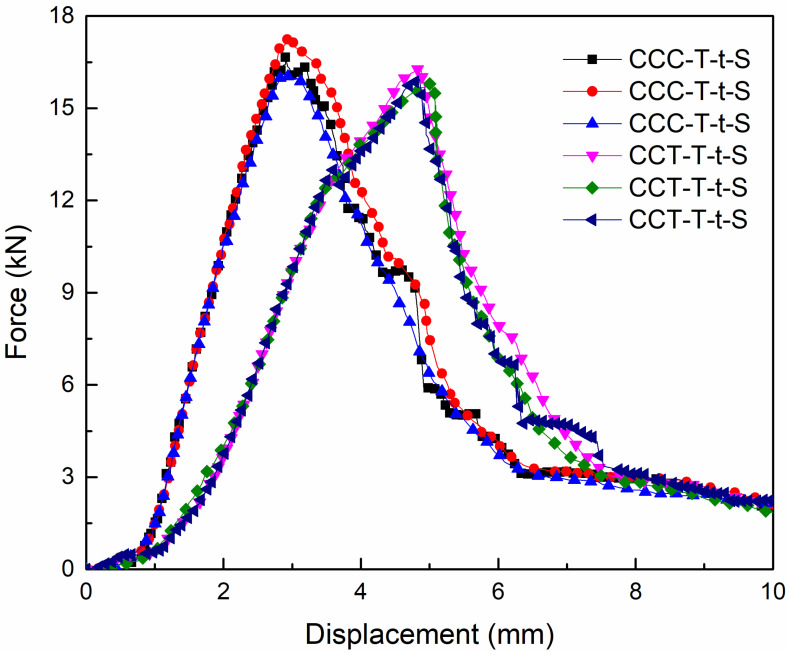
Tensile average load–displacement curves of two types of joints at room temperature.

Figure 7 presents the tensile load–displacement curves of CCC-T-t-S and CCT-T-t-S specimens under room-temperature conditions, with maximum tensile failure loads of 17.28 kN and 16.37 kN, respectively. The data presented are from three specimens within the same group. The average shear failure strengths for both types of joints were found to exceed 6.3 MPa. In this study, the shear failure strength of the adhesively bonded tube joint can be determined using the following formula: τ=F/S, where F represents the ultimate failure load, and S denotes the adhesive bonding area between the composite tube and the titanium alloy tube.

The influence of the spew-fillet on the lap strength was negligible. Moreover, the load–displacement curve exhibited an approximately linear behavior and a three-stage progression. During the initial stage, the tensile load increased swiftly with the rise in displacement. In the second stage, as the tensile displacement continued to grow, the ultimate load experienced a sudden and sharp increase, culminating in the attainment of the maximum failure load limit. In the final stage, the load declined rapidly while the displacement increased significantly. Concurrently, failure occurred in the adhesive layer or fiber, resulting in the joint’s loss of load-bearing capacity.

### 3.2. Influence of Failure Modes at Low Temperature

The effect of both ideal adhesive bonding conditions and the presence of a spew-fillet on the adhesively bonded strength of axial single-lap joints was assessed at a temperature of −65 °C. The test specimens were placed inside a temperature-controlled chamber. Liquid nitrogen at −196 °C was introduced into the chamber using a cryogenic Dewar, and the temperature within the environment box was steadily reduced and maintained at −65 °C for 15 min, in accordance with the procedures outlined in ASTM D5868-95 [42].

**Figure 8:**
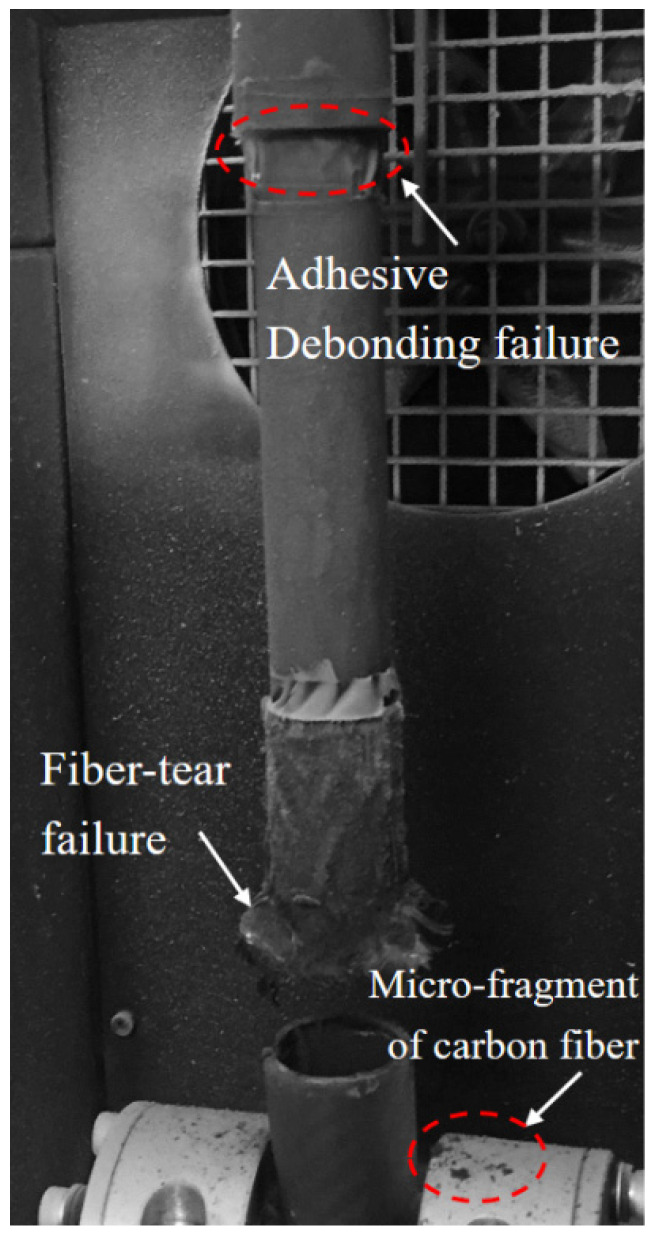
Final failure morphology of the CCC-T-t-S joint at −65 °C.

**Figure 9:**
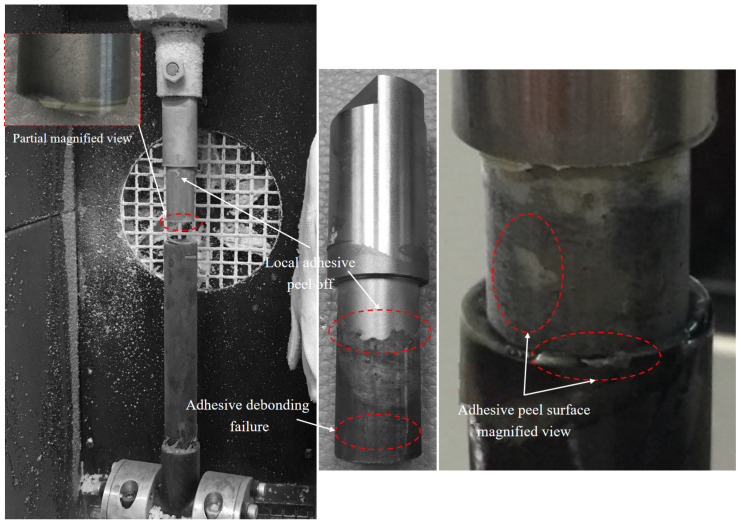
Final failure morphology of the CCT-T-t-S joint at −65 °C.

Figure 8 illustrates the final failure morphology of the CCC-T-t-S joint, characterized primarily by fiber-tear failure at one lap end of the carbon fiber tube, accompanied by adhesive debonding failure at the opposite end, along with the presence of carbon fiber micro-debris. Observations revealed that the carbon fiber three-way tube at the specimen’s bottom end initially underwent fiber-tear failure, followed by adhesive debonding failure in the carbon fiber three-way tube at the upper end. Figure 9 depicts the final failure morphology of the CCT-T-t-S joint. It was observed that the titanium alloy tube at the specimen’s upper end experienced adhesive debonding failure first, while the carbon fiber three-way tube at the bottom end remained largely undamaged. The carbon fiber three-way tube showed minimal damage, whereas the titanium alloy tube predominantly exhibited adhesive debonding failure. Local adhesive peeling was also noted on the titanium alloy tube wall, likely due to stress concentration at the joint’s end.

**Figure 10:**
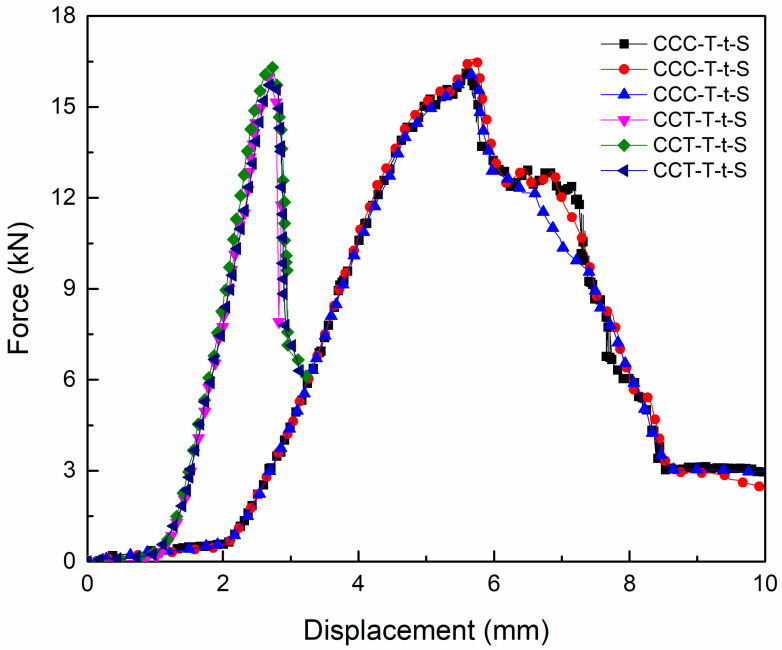
Tensile average load–displacement curves of two types of joints at low temperatures.

Figure 10 presents the tensile load–displacement curves of CCC-T-t-S and CCT-T-t-S specimens under low-temperature conditions. As depicted, the maximum tensile failure loads for the two joint types were 16.57 kN and 16.31 kN, respectively, with their average shear failure strengths surpassing 6.3 MPa. The failure displacement of the former was greater than that of the latter upon reaching the load limit. This discrepancy arises because the titanium alloy tube loses its load-bearing capacity immediately upon adhesive debonding failure, whereas fiber-tear failure constitutes a progressive damage process, allowing for sustained load-bearing capacity until final failure. Additionally, the influence of the spew-fillet on lap strength was minimal. Similar to behavior observed at room temperature, the load–displacement curve exhibited nearly linear characteristics, with the three-stage evolution being more pronounced in the CCC-T-t-S joint compared to the CCT-T-t-S joint.

### 3.3. Influence of Failure Modes Under Room–Low–Room-Temperature Cycling

To explore the specific applicability of adhesively bonded joints in the lightweight carbon fiber pod structure of near-space aerostat, the effects of both perfect lap and spew-fillet configurations on the load–displacement curve of titanium alloy axial single-lap joints were investigated under room–low–room-temperature cycling conditions. Uniaxial tensile failure tests were performed on two groups of test specimens: one group with a perfect lap configuration (Figure 2) and the other with a spew-fillet (Figure 2). The testing procedure was as follows. First, a lead weight was attached to one end of the test specimen (Figure 3), and a pre-tension of 850 N was applied and maintained for 12 h in an environmental chamber. This included 1.5 h at room temperature, cooling to −65 °C, 9 h at −65 °C, and 1.5 h warming back to room temperature. Subsequently, the specimen was removed and kept at room temperature for 24 h. Finally, the specimen was mounted on a mechanical testing machine to conduct the tensile failure test.

**Figure 11:**
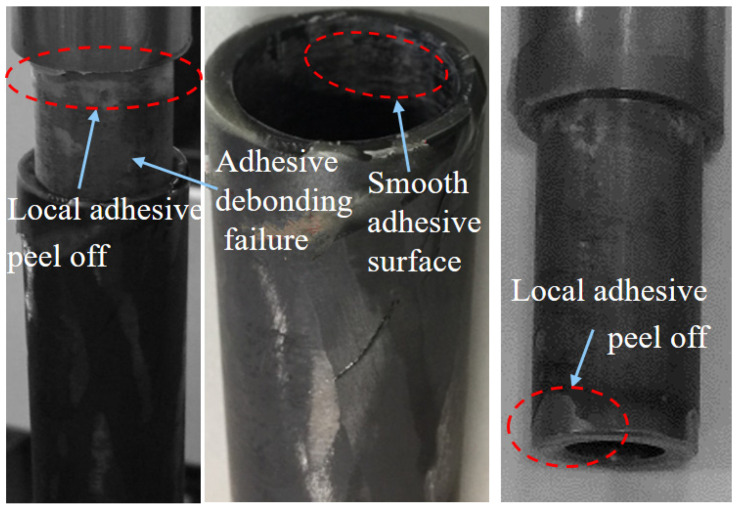
Final failure morphology of the TCT-T-t-P/S joint under room–low–room-temperature cycling.

Figure 11 illustrates the ultimate failure morphology of the TCT-T-t-P/S joint, predominantly characterized by adhesive debonding failure. Observations revealed that the titanium alloy tube at the specimen’s upper end experienced initial adhesive debonding failure, whereas the tube at the bottom end remained largely undamaged. The internal walls of both the carbon fiber circular tube and the titanium alloy tube exhibited a smooth adhesive surface. Consistent with the findings from the CCT-T-t-S test specimen, localized adhesive peeling was also evident on the titanium alloy tube.

**Figure 12:**
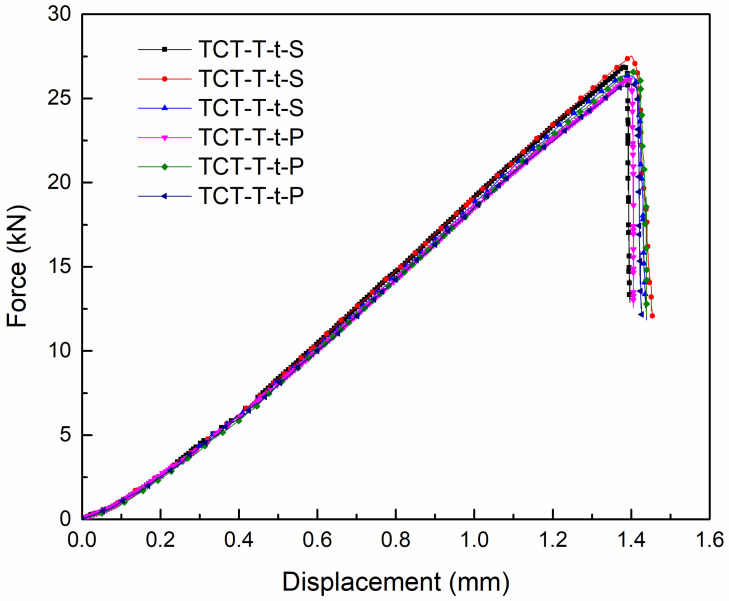
Tensile average load–displacement curves of two types of joints under room–low–room-temperature cycling.

Figure 12 presents the tensile load–displacement curves for the TCT-T-t-P and TCT-T-t-S test specimens, with maximum tensile failure loads of 26.57 kN and 27.52 kN, respectively. The shear failure strength exhibited a significant increase compared to that observed at room temperature or low temperature. Additionally, the tensile displacement at ultimate failure was lower than that recorded at room temperature. When compared to test results obtained at room or low temperatures, the bearing capacity under room–low–room-temperature cycling increased by about 10 kN, and the tensile failure limit rose by approximate 60%. This enhancement is likely attributed to the differential thermal expansion behaviors of the composite materials and titanium alloy. Specifically, under low-temperature conditions, the composite materials expand while the titanium alloy contracts. Following the thermal cycling test in the environmental control box, the composite circular tube contracts under normal temperature conditions, whereas the titanium alloy expands. This mismatch in the coefficients of thermal expansion between the two materials under varying temperature cycling conditions likely contributed to the increased bonding strength between the adherend and the adhesive layer.

The load–displacement curve exhibited approximately linear characteristics, but unlike the three-stage evolution observed under room- or low-temperature conditions, it did not follow a similar progression. This is because once adhesive layer debonding failure occurred, the adhesively bonded joint immediately lost its bearing capacity. This phenomenon is marked by a sharp, cliff-like drop in the load–displacement curve as soon as the failure load reaches its peak.

## 4. Analysis of Failure Load and Lap Shear Strength

**Figure 13:**
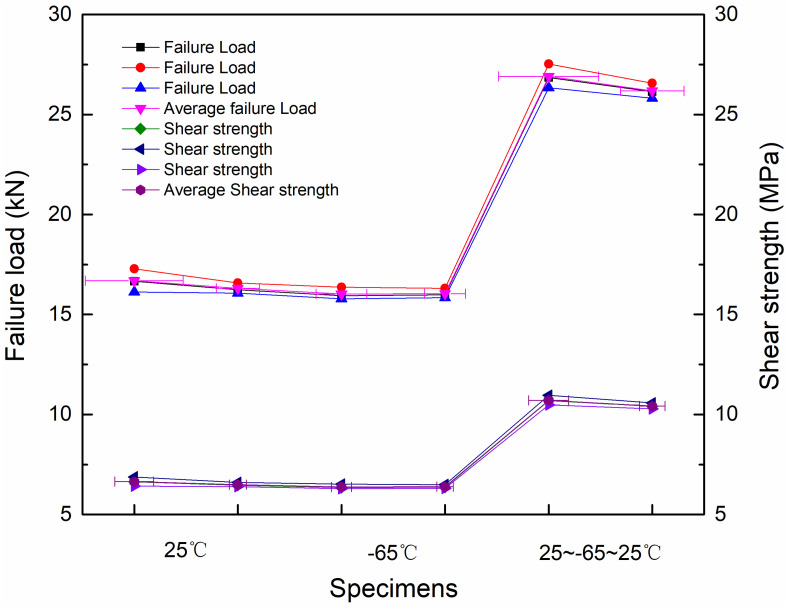
The ultimate failure load and shear strength of different types of joints under different temperature conditions.

Figure 13 presents a comparison of the average failure load and shear strength for the three adhesively bonded joints under different temperature conditions. The bonding strength was tested across a temperature range of −65 °C to 25 °C to validate the specific application of composite circular tube adhesively bonded joints in the lightweight pod structure of near-space aerostat. As illustrated, the maximum average failure load and shear strength were achieved under room–low–room-temperature cycling conditions. The influence of room-temperature or low-temperature conditions alone on the load limit and shear strength of the adhesively bonded joints was relatively minor. A comparison between the CCC-T-t-S and CCT-T-t-S joints revealed that, under identical temperature conditions, the presence of an adhesive spew-fillet had almost no effect on the ultimate failure load or shear strength of the joints. However, the ultimate failure load and shear strength of the adhesively bonded joints under room–low–room-temperature cycling conditions were significantly higher than those under constant room-temperature or low-temperature conditions. This suggests that the performance of the adhesively bonded joints can be substantially enhanced after undergoing a −65 °C low-temperature cycling process. This improvement is likely attributable to the mismatch in the thermal expansion coefficients of carbon fiber and titanium alloy, which enhances the bonding strength of the joint.

A reasonable explanation for the aforementioned phenomena is as follows: temperature variations can significantly alter the mechanical properties, surface tension, and surface energy of materials, which in turn may influence the adhesive bonding effectiveness. For instance, elevated temperatures can degrade the mechanical properties of materials, thereby reducing adhesive strength. Conversely, at lower temperatures, alterations in surface tension and surface energy may enhance the interaction between the adhesive and the bonded materials. During the room-to-low-to-room-temperature cycling process, the surface tension and surface energy of materials undergo changes. Carbon fiber tubes exhibit excellent stability at low temperatures, with a linear expansion coefficient ranging between 1.5 × 10^−6^/°C and 3.0 × 10^−6^/°C. In contrast, TC4 titanium alloy has a linear expansion coefficient between 8.6 × 10^−6^/°C and 9.8 × 10^−6^/°C, which minimally affects its surface tension. Epoxy resin, with a linear thermal expansion coefficient of approximately 50 × 10^−6^/°C and a large bonded area, experiences significant impacts on its surface tension. After cycling from low to room temperature, the linear thermal expansion coefficient of epoxy resin increases to about 70 × 10^−6^/°C, leading to a substantial rise in its surface tension and surface energy. This change in the thermal expansion coefficient facilitates a stronger interaction between the adhesive and the bonded materials, significantly enhancing the adhesive force between titanium alloy and carbon fiber tubes. Macroscopically, this results in a notable increase in the adhesive bonding load-bearing capacity.

Table 3 presents the failure load and shear strength of four different test specimens (CCC-T-t-S, CCT-T-t-S, TCT-T-t-P, and TCT-T-t-S) under varying temperature conditions. As shown in the table, the average failure load of CCC-T-t-S and CCT-T-t-S at both room temperature and low temperature (−65 °C) does not exceed 17 kN, with an average shear strength below 7 MPa and a maximum standard deviation of no more than 0.5. This indicates that temperature has a minimal effect on the failure load and shear strength of these specimens. In contrast, under environmental simulation conditions (25 °C to −65 °C to 25 °C), the average failure load and shear strength of TCT-T-t-S and TCT-T-t-P do not exceed 27 kN and 11 MPa, respectively, with the maximum standard deviation also remaining below 0.5. Compared to the results at room temperature and low temperature, the failure load and shear strength under environmental simulation conditions show a significant increase, with growth rates of 61.24% and 61.25%, respectively. This suggests that the bearing capacity of the circular tube bonding is substantially enhanced after undergoing room–low–room-temperature cycling.

## 5. Conclusions

In this study, the mechanical behavior of adhesively bonded single-lap joints between T700/C204 carbon fiber-reinforced epoxy resin composite circular tubes and TC4 titanium alloy tubes was investigated. The following conclusions were drawn:

1. Under both room-temperature and low-temperature conditions, the temperature had minimal impact on the tensile failure load of the joints. At room temperature, the primary failure mode observed in the carbon fiber three-way tube was fiber-tear failure. In contrast, at low temperatures (−65 °C), the titanium alloy tube predominantly exhibited adhesive debonding failure at one end of the specimen, while the carbon fiber three-way tube primarily showed fiber-tear failure at the opposite end. The presence of a spew-fillet at the end of the lap region in the adhesive did not significantly enhance the strength of the adhesively bonded joints. Notably, severe shear stress concentration was observed at the end of the lap region across various types of adhesively bonded joints, marking the initial point of joint failure.
2. Under simulated conditions of room temperature to low temperature and back to room temperature, the adhesive bonding between the carbon fiber circular tube and the titanium alloy joint achieved the maximum load-bearing capacity 27.522 kN and the highest shear strength 10.956 MPa. The failure mode of the adhesively bonded joints primarily exhibited irregular adhesive interface debonding. These specimens achieved higher failure loads and bonding strengths compared to other groups after undergoing room–low–room-temperature cycling. The tensile failure limit increased by approximately 61% relative to that observed under room-temperature or low-temperature conditions alone; however, the average tensile failure displacement remained relatively low. Following thermal cycling, the bonding strength of the single-lap joints was significantly enhanced.

## Figures and Tables

**Table 1:** Component material properties of specimens.

Materials	Properties			
T700/C204	*E*_1_ = 130 GPa	*G*_12_ = 5.0 GPa	*ν*_12_ = 0.40	*ρ* = 1.65 g/cm^3^
	*E*_2_ = 9.1 GPa	*G*_23_ = 4.3 GPa	*ν*_23_ = 0.38	
	*E*_3_ = 9.1 GPa	*G*_31_ = 5.0 GPa	*ν*_31_ = 0.02	
TC4	*E* = 110 GPa	-	*ν* = 0.34	*ρ* = 4.5 g/cm^3^
	*σ* = 1100 MPa			
J-250	*E* = 1 GPa	-	*ν* = 0.3	*ρ* = 0.6 g/cm^3^
	*σ* = 24.5 MPa	*τ* = 25 MPa		

**Table 2:** Details of test specimens.

Specimens	Ply Sequences	Adherend Thickness (mm)	Adhesive Thickness (mm)	Single-Lap Forms	No. of Specimens
CCC-T-t-S	[0/±45/0]_2S_	2.5/2.5	0.15	Adhesive spew-fillets (25 °C)/ Adhesive spew-fillets (−65 °C)	3/3
CCT-T-t-S	[0/±45/0]_2S_	2.5/4.0	0.15	Adhesive spew-fillets (25 °C)/ Adhesive spew-fillets (−65 °C)	3/3
TCT-T-t-P/S	[0/±45/0]_2S_	2.5/4.0	0.15	Perfect lap (25~−65~25 °C)/ Adhesive spew-fillets (25~−65~25 °C)	3/3

**Table 3:** Failure load and shear strength test results.

Specimens	Failure Load/kN			Average Failure Load/kN	Standard Deviation	Shear Strength/MPa			Average Shear Strength/MPa	Standard Deviation
CCC-T-t-S (25 °C)	16.655	17.277	16.124	16.686	0.471	6.630	6.878	6.419	6.642	0.188
CCC-T-t-S (−65 °C)	16.237	16.572	16.068	16.311	0.209	6.464	6.597	6.397	6.486	0.083
CCT-T-t-S (25 °C)	15.939	16.365	15.787	16.030	0.245	6.345	6.515	6.285	6.382	0.097
CCT-T-t-S (−65 °C)	15.976	16.315	15.847	16.046	0.197	6.360	6.495	6.309	6.388	0.078
TCT-T-t-S (25 °C~−65 °C~25 °C)	26.852	27.522	26.340	26.905	0.484	10.690	10.956	10.486	10.710	0.193
TCT-T-t-P (25 °C~−65 °C~25 °C)	26.145	26.571	25.821	26.179	0.307	10.408	10.578	10.279	10.422	0.122

## Data Availability

The original contributions presented in this study are included in the article. Further inquiries can be directed to the corresponding author.
